# Dr. Hawker, on Ophthalmia

**Published:** 1804-11-01

**Authors:** Peter Hawker

**Affiliations:** Tullamore, Ireland


### *To the Editors of the Medical and Physical Journal.*

GENTLEMEN,

LOOKING over a vol. of your very interesting Journals the other day, my attention was arrested by a paper on the Ophthalmia of hot climates, by a gentleman signing himself D. Whyte, M. D. whom I take to have been Surgeon, or Mate, to one of the men of war then lying in Aboukir Bay. I think it is contained in No. 37, dated March, 1802. He there very gravely informs his readers, that nature has most providentially supplied the eyes with eyelids for their protection, and with tears for the purpose of moistening them. He attributes inflammation of the eyes generally to "expansion of the humours and dilatation of the vessels from the intense heat and vivid rays of the sun, and accounts for the imperfect vision accompanying this species of inflammation, (in which species, however, he says the inflammation is not at all perceptible) from the alteration of the medium through which the rays of light pass. He instances a case of an acquaintance of his, who fell asleep on the rock of Gibraltar, with his eyes wide open, who recovered, notwithstanding, without the aid of a surgeon. The Doctor laments that this person had not put himself under his care, that he might have had an opportunity of assisting nature, by frequent applications of astringent lotions, and by *tapping the aqueous humour*, and reducing its expanded bulk to the standard quantity, sufficient to collect the rays of light into a focus on the retina. Being no doubt a skilful operator, he entertains no apprehension of suffering more to escape than might be necessary; but it does not seem to occur to him, that when, by the use of his styptic lotions, the tunics, with their contents, were reduced to their natural bulk and capacity, the portion he had drawn off would be wanting to direct the condensed rays to their usual focus.

This affection of the eyes, or, according to Dr. Whyte's definition, this "imperceptible" kind of inflammation, is nothing more than what every body experiences, in a lesser degree, in coming from a very light room into a dark apartment. The organ of vision, accustomed to the continued glare of the sun during the day, is not acted upon by the faint light of the night, and requires no other treatment than that the patient remain in a darkened apartment for a couple of days, when the functions of the eye are perfectly restored. Soldiers and sailors, who are much exposed during the day, are very subject to this inconvenience, on their first arrival in a hot climate; but I have never heard it complained of by troops, who had been resident there for any length of time; nor are the negroes at all subject to it, though they are exposed with their heads uncovered all day long at their field labour.

The next species the Doctor takes notice of is, "when the vessels of the tunica albuginea have alone suffered," means no doubt a real inflammation of the external tunics of the eye. He places his chief dependance on astringent lotions and styptic tinctures, applied with a hair pencil to the eyes, and, according to circumstances, leeching, opening the veins, arteries, &c. which, no doubt, the use of his astringents and styptics would, in most cases of active inflammation, produce a necessity for. He does not here approve of scarification; but in the next page he is so much an advocate for it, that he condemns the French physicians for not adopting it, and presumes to suppose that they know nothing at all about ocular inflammation. He takes occasion in this place to suppose, that could his tonics and astringents be applied to internal inflammations, a universal panacea might be discovered! Now I do not see what might prevent the doctor from confirming this ingenious theory, by injecting into an inflamed bladder some corrosive sublimate, dissolved in ardent spirit, or any of his stimulating tinctures. Or, in case of gastritis or enteritis occurring in the course of his practice, he might drench his patients with similar draughts. I do not by any means agree with the Doctor, that those inflammations are produced by heat and light alone, for in the hottest situations, where the temperature of the day and night is uniform, they are not found to be frequent; but prevail chiefly in places that experience a considerable difference in this particular. That this is the case on board ships lying in harbour, in tropical climates, climates, is well known; and I am told that the climate of Egypt is remarked for the difference of temperature between the days and nights. The reflection of heat and light from whitened squares and streets, or from light sandy beaches, is no doubt a considerable cause of ocular inflammation. My personal, as well as general practical experience, leads me entirely to differ from the Doctor, and substitute mild, cooling, subacid applications, such as the pulp of fruits, or scraped potatoe, to his tonics and stimulants, during the inflammatory stage; when that is passed, a mild astringent wash, much diluted, may perhaps be used with advantage; I have found cold water most beneficial. The eyes must be protected from the breeze, as well as from intense heat and light, for a considerable time after the cure.

I am, &c.

*Tullamore, Ireland, Aug.* 18, 1804. PETER HAWKER.

P. S. In your number for May, 1802, I find some cases of a disease by Mr. Chevalier, which he denominates catarrhus suffocativus, or coryza trachealis. In relating the history of those cases, it is singular that he does not take the smallest notice of the state of the trachea until after death, though he dwells on the state of the head and thorax, particularly as he found in each case, after dissection, that the trachea had been inflamed, and had indeed been the sole seat of the disease. A case of this kind occurred to me about four years since. An old gentleman, whose constitution was much debilitated, from a life spent in every kind of excess, complained to me one morning of a sore throat, attended with considerable pain. On examination, I found the glands and fauces somewhat inflamed; I prescribed some opening medicine, and the steam of hot water and vinegar to be inhaled twice or thrice in the day. About two o'clock the next morning I was sent for, and found him sitting up in his bed, unable to lie down from pain and difficulty of breathing, and looking exceedingly wild and alarmed. He was unable to speak, and his respiration was so sonorous that it might be heard at the hall door. I ordered some blood to be taken from him, and a blister to be applied to his throat; but it was too late, he died in half an hour. Had I seen this gentleman in the evening, and discovered the affection of his trachea, which was so obvious the next morning, a blister would in all probability have saved his life.

